# Spectroelectrochemical Determination of Isoprenaline in a Pharmaceutical Sample

**DOI:** 10.3390/s20185179

**Published:** 2020-09-11

**Authors:** Fabiola Olmo, Jesus Garoz-Ruiz, Julia Carazo, Alvaro Colina, Aranzazu Heras

**Affiliations:** Department of Chemistry, Universidad de Burgos, Pza. Misael Bañuelos s/n, E-09001 Burgos, Spain; folmo@ubu.es (F.O.); jgarozruiz@ubu.es (J.G.-R.); jcc0080@alu.ubu.es (J.C.); acolina@ubu.es (A.C.)

**Keywords:** spectroelectrochemistry, electrochemistry, UV/Vis absorption, drug analysis, isoproterenol, metabisulfite

## Abstract

UV/Vis absorption spectroelectrochemistry (SEC) is a multi-response technique that has been commonly used for the characterization of materials and the study of reaction mechanisms. However, it has been scarcely used for quantitative purposes. SEC allows us to obtain two analytical signals simultaneously, yielding a dual sensor in just one experiment. In the last years, our group has developed new devices useful for analysis. In this work, a SEC device in parallel configuration, based on optical fibers fixed on screen-printed electrodes, was used to determine isoprenaline in a commercial drug, using both, the electrochemical and the spectroscopic signals. In this commercial drug, isoprenaline is accompanied in solution by other compounds. Among them is sodium metabisulfite, an antioxidant that strongly interferes in the isoprenaline determination. A simple pretreatment of the drug sample by bubbling wet-air allows us to avoid the interference of metabisulfite. Here, we demonstrate again the capabilities of UV/Vis absorption SEC as double sensor for analysis and we propose a simple pretreatment to remove interfering compounds.

## 1. Introduction

Adrenergic receptors are targets of different catecholamines stimulating the sympathetic nervous system. Isoprenaline or isoproterenol (IP) is a non-selective sympathomimetic β-adrenergic agonist drug that acts on the β-adrenoreceptors. β-adrenergic agonists imitate the behavior of neurotransmitters that belong to the catecholamine family such as adrenaline or noradrenaline. IP is an important catecholamine drug, which is widely used for the treatment of neural disorders, highlighting Parkinson’s disease, heart attack, cardiogenic shock, bronchial asthma, allergic effects, and primary pulmonary hypertension. To adjust the suitable dosage of this drug and to avoid many side effects related to excessive IP consumption, such as necrotic lesion in heart, arrhythmias, and heart failure, it is essential to develop analytical methods to quantify IP in a simple and fast way in pharmaceutical samples or biological fluids [1,2,3,4,5,6,7].

Several methods have been used to quantify IP such as chemiluminescence [8,9], chromatographic-mass spectrometry (LC-MS) [10], chromatography [11], spectroscopy [12], or flow injection analysis [13,14]. However, the low sensitivity and the large amount of time required to carry out these analyses have led to the development of several electrochemical methods [1,2,3,7,15,16]. The final goal of all these methods is the determination of IP in complex matrices or in the presence of different potential interfering species [2,3,5,6,16]. Many electrochemical methods proposed to quantify IP require the modification of the electrode surface to achieve the sensitivity and/or selectivity needed. Different materials, such as polymers [15] or nanostructures like multiwalled carbon nanotubes [3,5,17,18], graphene [7], hollow carbon spheres [16], or iridium oxide nanoparticles [19], have been proposed to modify the electrode surface. Recently, an electrochemical sensor which contains lanthanum doped zinc oxide and reduced graphene oxide has been described for the selective determination of IP overcoming interference problems [6].

In this work, we propose for the first time to the best of our knowledge, the determination of IP with spectroelectrochemistry (SEC). This time-resolved multi-response technique has allowed the quantification of different molecules in a simple and fast way, even in complex matrices, without using modified electrodes and resolving high interfering mixtures [20,21,22,23]. SEC provides in a single experiment two independent signals related to the same chemical system, one electrochemical and one spectroscopic [24,25,26,27]. These two signals can be used to determine an analyte, emerging as a self-validated analytical technique [23]. 

UV/Vis absorption SEC experiments have been carried out to study and to determine IP. Two different optical configurations can be used to perform this type of analysis, the so-called normal and parallel configurations [25,26,28,29,30,31]. The experiments carried out in normal configuration provide information related to the processes that take place on the surface of the electrode, such as adsorption processes, and in the solution adjacent to it, such as the oxidation or reduction of a soluble molecule. To achieve this objective a light beam hits the electrode surface in a perpendicular direction. If the electrode is optically transparent, the electromagnetic radiation passes through the electrode and the transmitted light beam is collected. If the electrode is opaque, the light beam is reflected on the electrode surface. The optical path-length in normal configuration is very short in the order of micrometers, coinciding with the thickness of the diffusion layer. On the other hand, in experiments carried out in parallel configuration, the light beam passes parallel to the electrode surface allowing us to interrogate only the first micrometers of the solution adjacent to the working electrode and to detect all spectral changes in solution related to the electron-transfer reaction occurring at the electrode surface. All experiments carried out in this work have been performed selecting the parallel configuration because it provides better sensitivity for soluble analytes than the normal configuration, due to the longer optical path-length [24,30]. It should be noted that in parallel configuration the length of the optical path-length can be as long as the length of the electrode surface, which can be in the order of millimeters.

Commercial screen-printed electrodes (SPEs) and two bare optical fibers suitably aligned and fixed on the electrode surface [21] are enough to carry out the analysis proposed in this work. IP can be properly determined using a fixed optical pathway controlled by the distance between the ends of the two optical fibers attached on the electrode surface. Particularly, IP has been quantified in a pharmaceutical vial of isoprenaline sulphate, commercially known as Aleudrine, in which sodium metabisulfite is the main interfering species. Metabisulfite is an antioxidant compound used as preservative in some foods and drugs [32,33]. For example, sodium metabisulfite is present in IP pharmaceutical products with the aim of minimizing its instability and avoiding its degradation [33]. Due to its antioxidant properties, metabisulfite greatly inhibits the IP oxidation avoiding its proper electrochemical determination in pharmaceutical samples. In this work, we propose a simple methodology to overcome this drawback, being able to determine IP without the need of using modified electrodes or tedious and time-consuming techniques or procedures.

## 2. Materials and Methods

### 2.1. Reagents and Materials

Isoprenaline hydrochloride (98%, Acros Organics) was used as received without further purification. All solutions were daily prepared at room temperature, in high-quality water (18.2 MΩ cm resistivity at 25 °C, Milli-Q Direct 8, Millipore) and with 0.1 M HCl (37%, VWR) as supporting electrolyte. Isoprenaline sulphate 0.2 mg/mL, whose pharmaceutical name is Aleudrine (LAB. REIG JOFRE, S.A.), was selected as the real sample.

### 2.2. Instrumentation and Software

A customized SPELEC instrument (Metrohm-DropSens), controlled by DropView SPELEC software (Metrohm-DropSens), was used to perform SEC experiments. Our group in collaboration with Metrohm-DropSens has developed this instrument. Matlab R2018a and R x64 3.6.2 were used to carry out the data processing.

### 2.3. Experimental Setup

**Figure 1:**
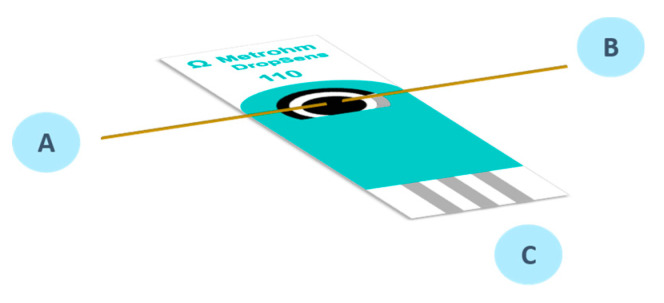
Scheme of the screen-printed electrode (SPE) with the optical fibers used to register the UV/Vis SEC changes. A is the optical fiber connected to the light source, B is the optical fiber connected to the spectrometer, and C is the link of the three electrodes with the potentiostat.

The device used in this work to carry out UV/Vis absorption SEC experiments was previously described by our research group [21]. In this case carbon pristine SPEs (DRP-110, Metrohm-DropSens), which includes a 4-mm diameter disc carbon working electrode (WE), a carbon counter electrode (CE), and a silver pseudo-reference electrode, were used for all SEC measurements. Two bare optical fibers (100 μm in diameter, Ocean Optics) were aligned on the working electrode using nail polish to fix them. In this way, SEC experiments can be carried out in parallel configuration (Figure 1), where the optical fiber A was connected to the light source, and the optical fiber B was connected to the spectrophotometer to collect the light transmitted by the sample. Simultaneously, the SPE registers the electrochemical changes through the connection C that was connected to the potentiostat. A solution drop (50 μL) was placed on the electrode, covering the three-electrode system and the ends of the two bare optical fibers. In all experiments, the spectrum of the initial solution (IP in 0.1 M HCl) at the starting potential (−0.10 V) was taken as reference spectrum to subsequently calculate the absorbance values. 

## 3. Results and Discussion

### 3.1. Spectroelectrochemical Behavior of Isoprenaline

**Figure 2:**
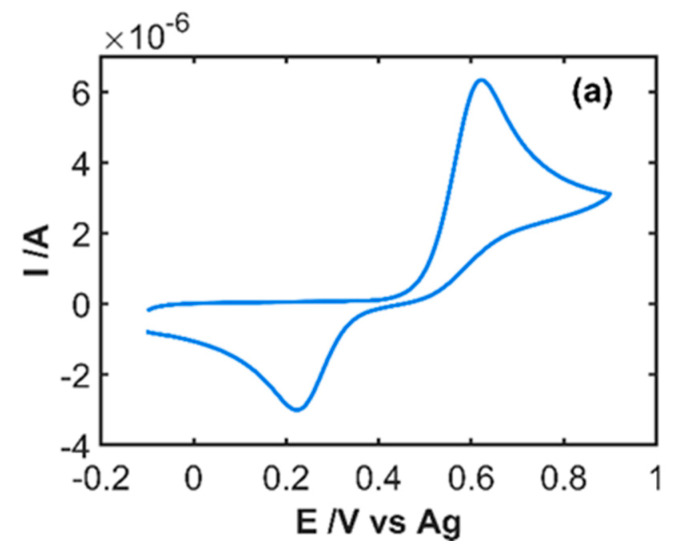

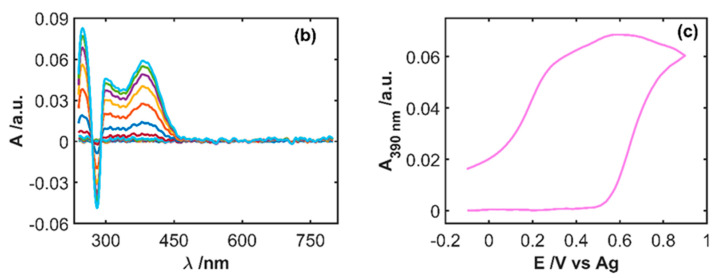
Spectroelectrochemistry (SEC) experiment of 5 × 10^−4^ M IP in 0.1 M HCl between −0.10 and +0.90 V at 0.01 Vs^−1^. (**a**) Cyclic voltammogram, (**b**) spectra evolution during the anodic scan, and (**c**) cyclic voltabsorptogram at 390 nm.

The first step carried out in this work was the study of the electrochemical oxidation process of IP, registering simultaneously the cyclic voltammogram and all spectral changes in the UV/Vis spectral region. Figure 2a shows the cyclic voltammogram of a solution 5 × 10^−4^ M IP in 0.1 M HCl recorded between −0.10 and +0.90 V at 0.01 Vs^−1^. A quasi-reversible electron transfer process is observed where an anodic peak emerges at +0.62 V and a cathodic peak at +0.22 V. 

**Figure 3:**
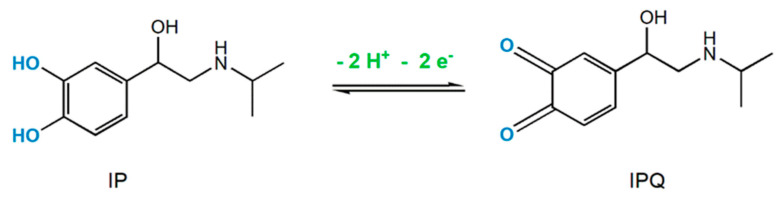
Redox mechanism of isoprenaline in acid medium (pH = 1).

As other catecholamines, in the forward scan IP is oxidized in acid medium to the corresponding quinone, known as isoproteroquinone (IPQ), in a process that involves the transfer of two protons and two electrons (Figure 3) [7,17]. Subsequently, in the backward scan, electrogenerated IPQ is reduced to IP.

Simultaneously to the cyclic voltammogram, the evolution of the absorption spectra between 210 and 1000 nm was registered every 500 ms. Thus, all spectral changes with respect to the starting IP solution were observed, providing absorptiometric information related to the oxidation process. Figure 2b shows the spectral changes during the forward scan between −0.10 and +0.90 V at selected potentials. Two absorption bands centered at 250 and 390 nm increase during IP oxidation due to the electrogeneration of IPQ, while an absorption band decreases at 280 nm related to the consumption of IP on the solution adjacent to the electrode surface. Two isosbestic points are clearly observed during anodic and cathodic scans, confirming the mechanism shown in Figure 3 [7,17], IPQ being the only oxidation product. The corresponding cyclic voltabsorptogram at 390 nm (Figure 2c), where absorbance at this characteristic wavelength of IPQ is plotted with respect to the applied potential, shows the increase of absorbance between +0.50 V, where IP oxidation starts, and the vertex potential (+0.90 V). At the beginning of the backward scan, the absorbance slightly increases because the potential is high enough to electrogenerate more IPQ that moves to the diffusion layer. When the potential at which IPQ is reduced to IP is reached, the absorbance starts to decrease due to the consumption of the IPQ previously electrogenerated. The correlation between the information supplied by the two signals, voltammogram and absorption spectra, indicates that both explain the electrochemical oxidation of IP. 

### 3.2. Determination of Isoprenaline Spectroelectrochemically

**Figure 4:**
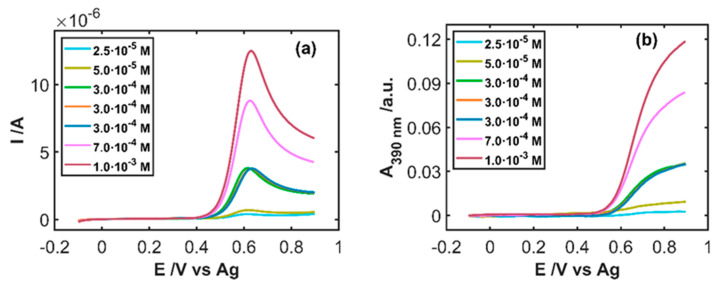
SEC experiments of different samples of IP in 0.1 M HCl between −0.10 and +0.90 V at 0.01 Vs^−1^. (**a**) Linear sweep voltammograms and (**b**) linear voltabsorptograms at 390 nm.

Once the behavior of IP was explained, a set of UV/Vis absorption SEC experiments were carried out to evaluate the correlation of these signals with the concentration of IP. Therefore, a set of nine calibration samples with different concentrations of IP between 5 × 10^−6^ and 1 × 10^−3^ M in HCl 0.1 M were measured. Linear sweep voltammograms were recorded between −0.10 and +0.90 V at 0.01 Vs^−1^ and, concomitantly, full absorption spectra were registered every 500 ms. Figure 4a shows the linear sweep voltammograms and Figure 4b the linear voltabsorptograms at 390 nm for some selected concentrations. As expected, an increase of the anodic peak current with the concentration is observed, as well as an increase of the absorbance values at this wavelength from +0.50 V onwards. 

**Figure 5:**
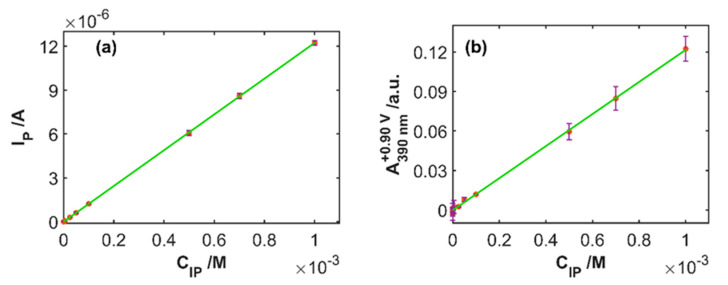
Calibration plots of the (**a**) current peak vs. IP concentration and (**b**) absorbance at 390 nm and at +0.90 V vs. IP concentration.

Linear calibration models using the electrochemical and the spectroscopic signals were constructed, considering that the measurements of the test samples which contains 3.0 × 10^−4^ M IP are not included. For each type of signal, the potential to obtain the highest sensitivity in the calibration models was chosen. Therefore, to construct these linear calibration models, IP concentration was correlated with the anodic peak current from the linear sweep voltammograms and with the absorbance values at 390 nm measured at the vertex potential (+0.90 V) from the spectral signals. Table 1 shows the figures of merit of the two linear calibration models obtained, considering that both were obtained from the same set of experiments. The calibration procedure was replicated three times using a different SPE in each one of the calibration sets. The optical pathway values, in this case equal to the distance between the optical fibers, were different in each SPE, therefore absorbance values were corrected with respect to one of them. Figure 5 shows the two calibration curves obtained, one related to the electrochemical data and the other one to the optical data. In the two cases, coefficients of determination close to 1 and low values of the standard deviation of the residuals (S_yx_) were obtained indicating the good linear relationship between these two variables and IP concentration. The limit of detection is lower for the electrochemical measurements (7 µM) than for the spectroscopic data (45 µM).

The reproducibility of the spectroelectrochemical methodology developed to quantify IP was demonstrated by performing three univariate calibration models with data collected on three different days for each kind of signal, electrochemical and spectroscopic. All solutions were also prepared daily and a different SPE was used each day. Optical fibers were fixed on different SPEs, changing the optical pathway between experiments carried out on different days. The optical pathway values vary between 0.19 and 0.17 cm. For this reason, each calibration model was corrected to properly compare the different slopes. The relative standard deviation (%RSD) of the slopes for the electrochemical models was 1.7%, being 9.8% for the spectroscopic models. These values indicate a good reproducibility of the spectroelectrochemical quantification of IP in 0.1 M HCl. 

The calibration models shown in Table 1 were used to predict the concentration of a test sample of 3.0 × 10^−4^ M IP in 0.1 M HCl. Table 2 includes the figures of merit related to this prediction, showing values very similar to the real concentration of IP with low relative error values. Furthermore, samples of 3 × 10^−4^ M IP in 0.1 M HCl were replicated nine times, which allowed us to evaluate the repeatability of the method. In these replicates, electrochemical and spectroscopic signals are very similar. Figure 4 shows three of these replicates appreciating that the current intensity and absorbance values reached are practically the same for these three measurements. The %RSD values (Table 2) are lower than 5%, indicating the good repeatability of the procedure proposed in this work.

Two very similar estimations of the concentration of the same sample are obtained in the same experiment, which significantly increases the reliability of spectroelectrochemistry in quantitative analysis.A test for comparison of the predicted concentrations obtained with the electrochemical and spectroscopic calibration models was also carried out. An intercept of [0 ± 8.8 × 10^−6^] and a slope of [1.00 ± 0.02] indicate the absence of bias, concluding that the two models can be used to estimate IP concentration indistinctively and demonstrating that spectroelectrochemistry is a self-validated analytical technique. This represents a great advantage in comparison with other analytical techniques commonly used to identify and quantify a specific analyte, in which only one analytical signal is obtained, and another analytical technique must be used to validate their results. 

The analytical performances of the UV/Vis absorption spectroelectrochemistry method developed in this work were compared with those obtained with other electrochemical and spectrophotometric methods specifically developed to determine IP (Table 3). Electrochemical methods very often involve the modification of the electrode surface. This leads to detection limits much lower than those obtained with cyclic voltammetry in the proposed spectroelectrochemistry technique. On the contrary, linear ranges are usually lower than those obtained in this work. Besides, electrode modifications are usually time consuming and long-term stability is not frequently as high as unmodified screen-printed electrodes. With respect to spectrophotometric methods, they are typically longer processes, and sometimes involve an interaction process or an intermediate reaction with another substrate, often making an indirect measurement of the presence of IP. Detection limits obtained are sometimes better than the one reached in our work, but the linear ranges are usually shorter in comparison with the one obtained here that covers three orders of magnitude. Additionally, when performing the determination of IP with spectroelectrochemistry, the electrochemical method validates the results of the spectrophotometric one and vice versa, automatically validating the results obtained as a whole. Moreover, changes in the spectra or in the voltammogram can help to easily identify anomalous samples.

### 3.3. Application of Spectroelectrochemistry Quantification to a Pharmaceutical Sample with Isoprenaline

Once the repeatability and reproducibility of the spectroelectrochemical procedure were demonstrated, its applicability was evaluated through the determination of isoprenaline sulphate in pharmaceutical vials of Aleudrine (LAB. REIG JOFRE, S.A.). This drug contains 0.2 mg/mL of isoprenaline sulphate, sodium metabisulfite as antioxidant agent, sodium edetate, sodium chloride, and hydrochloric acid. Each vial was diluted in 0.1 M HCl in a ratio of 19/100, the theoretical concentration of IP being equal to 1.46 × 10^−4^ M. 

**Figure 6:**
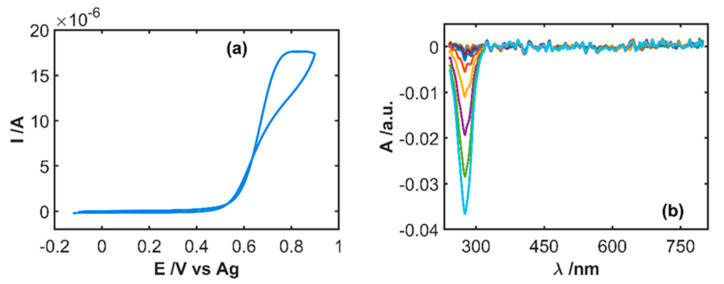
SEC experiment of the drug diluted 19/100 in 0.1 M HCl (1.46 × 10^−4^ M IP) between −0.10 and +0.90 V at 0.01 Vs^−1^. (**a**) Cyclic voltammogram and (**b**) evolution of UV/Vis absorption spectra during the anodic scan.

As was explained above, this pharmaceutical sample contains sodium metabisulfite, which is an interfering and antioxidant compound. In consequence, some influence on the IP oxidation was expected. Figure 6 shows the electrochemical and spectroscopic responses for a sample of the drug diluted in 0.1 M HCl. Many differences can be found with respect to the signals shown in Figure 2 for a pure IP sample, even when the experimental conditions are the same. Only one irreversible anodic peak evolves in the cyclic voltammogram (Figure 6a) peaking at +0.82 V, which is much higher than the anodic peak potential for the IP sample in Figure 2a. Furthermore, the peak current of this sample is three times higher than the signal shown in Figure 2a, although the IP concentration is 3.4 times lower. Changes occurring in the absorption spectra during the anodic scan, where IP should be oxidized, show only a band around 280 nm decreasing in intensity during oxidation of IP (Figure 6b). No evidence of electrogeneration of IPQ can be extracted from spectral signals because there are no bands centered at 250 and 390 nm. Therefore, the direct quantification of IP in this sample is not possible using this procedure.

**Figure 7:**
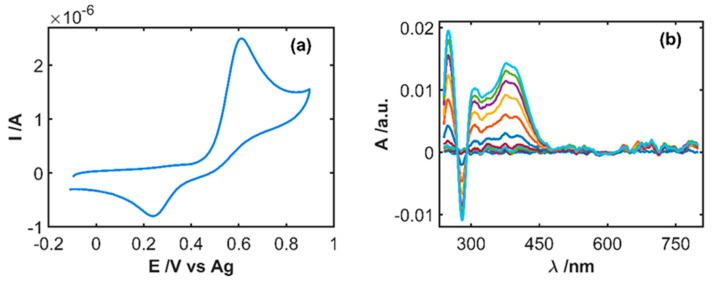
SEC experiment of the drug diluted 19/100 in 0.1 M HCl (1.46 × 10^−4^ M IP) between −0.10 and +0.90 V at 0.01 Vs^−1^, after bubbling wet-air for 5 min. (**a**) Cyclic voltammogram and (**b**) evolution of UV/Vis absorption spectra during the anodic scan.

To overcome this drawback, we propose a simple pretreatment of the sample consisting of the oxidation of metabisulfite by generation of sulfate to remove the interfering effect. The developed procedure consists of bubbling wet-air into the sample during a fixed time. The pretreated sample was measured following the same protocol described in Section 3.2. Figure 7 shows the results after bubbling wet-air for 5 min before performing the electrochemical oxidation of IP. Both, the cyclic voltammogram (Figure 7a) and the spectra evolution during the anodic scan (Figure 7b) are like the signals recorded for a test sample of 5 × 10^−4^ M IP in 0.1 M HCl (Figure 2). Again, a quasi-reversible electron transfer process is observed in the cyclic voltammogram, with an anodic peak at +0.61 V and a cathodic peak at +0.24 V, while two absorption bands evolve at 250 and 390 nm related to IPQ electrogeneration and one absorption band decreases at 280 nm due to the IP oxidation. Oxygen in air oxidizes metabisulfite to sulfate, but not IP to IPQ. Nevertheless, an optimization of the bubbling time was performed.

**Figure 8:**
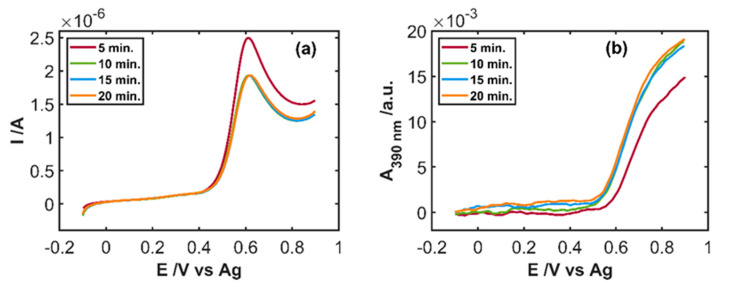
SEC experiment of the drug diluted 19/100 in 0.1 M HCl (IP 1.46 × 10^−4^ M) between −0.10 and +0.90 V at 0.01 Vs^−1^ after oxidation of sodium metabisulfite by fluxing wet-air for different times. (**a**) Linear voltammograms and (**b**) linear voltabsorptograms at 390 nm.

To optimize the time required to remove the antioxidant species four different set of experiments were carried out, bubbling the drug sample solution with wet-air for 5, 10, 15, and 20 min, after which the SEC experiment was performed. Figure 8 shows the linear voltammograms and the linear voltabsorptograms at 390 nm for these four experiments. Aleudrine samples were replicated showing the same results. The current intensity of the anodic peak related to the sample bubbled only for 5 min, is 30% higher than the intensity reached by the other three cases, measured after bubbling wet-air for 10, 15, and 20 min (Figure 8a). In addition, the voltammograms registered for samples after bubbling 10, 15, and 20 min are completely overlapped indicating that after 10 min the sample does not change any more. In the same way, the linear voltabsorptograms at 390 nm after metabisulfite oxidation by bubbling wet-air for 10, 15, and 20 min are very similar (Figure 8b). In fact, their absorbance values at +0.90 V are practically the same, but it is a 25% lower for the sample bubbled for only 5 min. These differences indicate that bubbling time affects the quantification of IP and it seems that 10 min of bubbling wet-air is enough to remove, by oxidation, all the sodium metabisulfite in the drug.

IP concentration was estimated with the calibration models shown in Table 1, and the results obtained from electrochemical and spectroscopic data are tabulated in Table 4, for the four different bubbling times. The relative errors obtained when the sample is oxidized for only 5 min are very high when they are compared with longer bubbling times, because not all metabisulfite is removed. Moreover, the great difference between the predicted IP concentration with the electrochemical and the spectroscopic models indicates that something is wrong in the analysis. This is a very nice example of the double information provided by SEC, which is very useful to detect anomalous situations. On the other hand, the quantification of IP for 10, 15, and 20 min is accurate, and the results are very similar for all these times, with relative errors lower than ±3.5%. IP concentration in the drug can be successfully determined with both the electrochemical and the spectroscopic signals after a simple pretreatment of the sample, by bubbling wet-air for at least 10 min. Similar results were obtained with voltammetric and voltabsorptometric data indicating that the suitable quantification of IP in a pharmaceutical sample with low %RSD values can be performed using spectroelectrochemistry.

## 4. Conclusions

SEC has demonstrated to be a suitable technique to study the IP oxidation process. An UV/Vis absorption spectroelectrochemical method was developed to quantify IP in acidic media. This methodology has demonstrated to provide a very good repeatability and reproducibility and the estimated IP concentration in test samples being very accurate. In addition, this technique was used to determine IP in a drug sample, Aleudrine. The pharmaceutical sample contained an interfering compound, sodium metabisulfite, to avoid the IP oxidation. Metabisulfite avoids the spectroelectrochemical quantification of IP in Aleudrine without a previous pretreatment of the sample. In this work, we proposed the oxidation of the interfering compound with a wet-air stream. After testing different oxidation times, 10 min was proposed as the optimal time to remove metabisulfite. In this way, a proper and accurate determination of IP in this drug can be performed by SEC. Both, electrochemical and spectroscopic results confirm that metabisulfite is removed after its oxidation with wet-air. IP was accurately estimated with low %RSD and relative error values in a simple and fast way, using a small sample volume and without modifying the electrode surface. 

The main advantage of SEC is that two signals of different nature are obtained in just one experiment, which is very useful both to quantify a specific analyte and to detect anomalous samples. In this case, both the electrochemical and the spectroscopic responses were successfully used to determine the IP present in the drug sample, demonstrating the self-validation of the results obtained by these two independent signals that are simultaneously registered. These results confirm that SEC could be easily implemented for routine quality control analysis.

## Figures and Tables

**Table 1:** Regression parameters obtained for the calibration models performed with electrochemical and spectroscopic data (*n* = 36).

Method *^(1)^*	Intercept *^(2)^*	Slope *^(3)^* (M^−1^)	R^2, *(4)*^	S_yx_ *^(5)^*	LOD *^(6)^*
I_P,an_	−2.52 × 10^−8^	1.22 × 10^−2^	0.9999	2.24 × 10^−8^	7.0 × 10^−6^
A_390 nm,+0.90 V_	−1.91 × 10^−4^	1.21 × 10^2^	0.9989	1.43 × 10^−3^	4.5 × 10^−5^

*^(1)^* Methods used to construct the calibration models. I_P,an_ correlates the anodic peak current of the voltammogram vs. IP concentration, and A_390 nm,+0.90 V_ correlates the absorbance at 390 nm and +0.90 V vs. IP concentration. *^(2)^* Intercept of the linear regression model. *^(3)^* Slope of the linear regression model. *^(4)^* Coefficient of determination. *^(5)^* Standard deviation of residuals. *^(6)^* Limit of detection.

**Table 2:** Estimation of the concentration of IP in a test sample.

Method	CI (M) *^(1)^*	%R.E. *^(2)^*	%RSD *^(3)^*
I_P,an_	[2.98 ± 0.04] × 10^−4^	0.64	0.5
A_390 nm,+0.90 V_	[2.98 ± 0.27] × 10^−4^	0.67	4.1

*^(1)^* Confidence interval. *^(2)^* Relative error calculated as the absolute value of {[Real]_IP_-[Estimated]_IP_/[Real]_IP_} × 100; [Real]_IP_ = 3 × 10^−4^ M. *^(3)^* Relative standard deviation of the concentration (*n* = 9).

**Table 3:** Comparison of analytical performances of the SEC proposed method and other electrochemical or spectrophotometric methods.

**Electroanalytical Techniques**	**Electrode**	**Linear Range** (**μM**)	**LOD** (**μM**)	**Ref.**
Amperometry	IrOxNPs/GCE	1–2500	0.09	[19]
LSV	HCSs/GCE	0.2–30	0.06	[16]
SWV	CuNPs-GO-CB-PEDOT:PSS/GCE	8.0–50	1.9	[2]
DPV	3% LZO/RGO/GCE	0.01–700	0.18	[6]
**Spectrophotometric techniques**	**Method principle**	**Linear range** (**μM**)	**LOD** (**μM**)	**Ref.**
Flow injection spectrophotometry using a color-forming reaction	Oxidation by polyphenol oxidase	123–738	63.4	[14]
Flow injection spectrophotometry using a solid-phase reactor	MnO_2_ immobilized in polyester resin	10–200	1.7	[34]
Spectrophotometry	Generation of a samarium-IP complex	9.5–57	7.6	[4]
Colorimetry	Interaction of IP and AMTD-AuNPs	0.2–2.6	0.08	[12]
**Absorption SEC**	**Electrode**	**Linear range** (**μM**)	**LOD** (**μM**)	**Ref.**
SEC (CV)	C-SPE	5–1000	7	This work
SEC (UV-Vis Spectra)			45	

LSV: linear sweep voltammetry; SWV: square-wave voltammetry; DPV: differential-pulsed voltammetry.

**Table 4:** Concentration estimated for the real sample of Aleudrine from regression curves tabulated in Table 2.

Time (Min) *^(1)^*	Method	CI (M) *^(2)^*	%R.E. *^(3)^*	%RSD *^(4)^*
5	I_P,an_	[2.05 ± 0.04] × 10^−4^	40.7	0.9
	A_390 nm,+0.90 V_	[0.99 ± 0.27] × 10^−4^	32.0	12.4
10	I_P,an_	[1.46± 0.04] × 10^−4^	0.2	1.3
	A_390 nm,+0.90 V_	[1.43 ± 0.27] × 10^−4^	2.2	8.6
15	I_P,an_	[1.47 ± 0.04] × 10^−4^	0.4	1.3
	A_390 nm,+0.90 V_	[1.49 ± 0.27] × 10^−4^	1.8	8.3
20	I_P,an_	[1.42 ± 0.04] × 10^−4^	2.5	1.3
	A_390 nm,+0.90 V_	[1.51 ± 0.27] × 10^−4^	3.5	8.1

*^(1)^* Time in minutes related to the fluxing time an air stream is passed through the drug sample. *^(2)^* Confidence interval. *^(3)^* Relative error calculated as the absolute value of {[Real]_IP_-[Estimated]_IP_/[Real]_IP_} × 100; [Real]_IP_ = 1.46 × 10^−4^ M. *^(4)^* Relative standard deviation of the concentration (*n* = 4).
